# G-Quadruplex Structures Formed by Human Telomere and *C9orf72* GGGGCC Repeats

**DOI:** 10.3390/ijms26041591

**Published:** 2025-02-13

**Authors:** Bing Yan, Monica Ching Suen, Naining Xu, Chao Lu, Changdong Liu, Guang Zhu

**Affiliations:** 1 State Key Laboratory of Molecular Neuroscience, Institute for Advanced Study, Division of Life Science, The Hong Kong University of Science and Technology, Clear Water Bay, Kowloon, Hong Kong SAR, China; byanab@connect.ust.hk (B.Y.); cmsuen@connect.ust.hk (M.C.S.); xunaining@ust.hk (N.X.); 2 HKUST Shenzhen Research Institute, Hi-Tech Park, Nanshan, Shenzhen 518057, China; 3 Department of Chemistry, Faculty of Science, The University of Hong Kong, Hong Kong SAR, China; luchaohk@connect.hku.hk

**Keywords:** G-quadruplex, telomere, neurodegenerative disease, *C9orf72*, ALS/FTD

## Abstract

G-quadruplexes (G4s) are unique nucleic acid structures composed of guanine-rich (G-rich) sequences that can form diverse topologies based on the arrangement of their four strands. G4s have attracted attention for their potential roles in various biological processes and human diseases. In this review, we focus on the G4 structures formed by human telomeric sequences, (GGGTTA)_n_, and the hexanucleotide repeat expansion, (GGGGCC)_n_, in the first intron region of the chromosome 9 open reading frame 72 (*C9orf72*) gene, highlighting their structural diversity and biological significance. Human telomeric G4s play crucial roles in telomere retention and gene regulation. In particular, we provide an in-depth summary of known telomeric G4s and focus on our recently discovered chair-type conformation, which exhibits distinct folding patterns. The chair-type G4s represent a novel folding pattern with unique characteristics, expanding our knowledge of telomeric G4 structural diversity and potential biological functions. Specifically, we emphasize the G4s formed by the (GGGGCC)_n_ sequence of the *C9orf72* gene, which represents the most common genetic cause of amyotrophic lateral sclerosis (ALS) and frontotemporal dementia (FTD). The thorough structural analysis in this review advances our comprehension of the disease mechanism and provides valuable insights into developing targeted therapeutic strategies in ALS/FTD.

## 1. Introduction

G-quadruplexes (G4s) represent remarkable four-stranded helical structures that emerge from G-rich nucleotide sequences, characterized by distinctively stacked two or more G-tetrad planes that comprise four guanines interconnected through Hoogsteen hydrogen bonds [1,2]. The structural integrity of G4s is preserved mostly by coordination through monovalent cations, typically, K^+^ and Na^+^, which occupy central ionic channels and confer necessary stabilization [3]. G4s can adopt several different conformations that can be classified as parallel, antiparallel, or hybrid types by the relative orientation of the strands [4]. Factors that influence this structural polymorphism include sequence composition, loop length, ionic conditions, and molecular crowding effects [5,6,7,8].

Currently, over 700,000 regions capable of forming G4 structures throughout the human genome have been extensively documented by computational and deep-sequencing approaches, which are notably enriched in functionally significant genomic regions, including promoters, telomeres, and regulatory elements [9,10,11]. The physiological relevance of G4 structures has been definitively established through multiple experimental approaches, including immunological detection, single-molecule fluorescence visualization, and small-molecule probing methodologies [12,13]. Accumulated studies have progressively unveiled the pivotal roles of G4s in biological processes including telomere retention, replication dynamics, transcriptional regulation, and genome stability [14,15,16].

Importantly, dysfunction in G4-mediated processes has been implicated in numerous pathological conditions, particularly various cancers and neurodegenerative diseases [17,18]. For example, the *C9orf72* hexanucleotide repeat expansion forms stable G4 structures linked to the neurodegenerative diseases ALS and FTD [17]. However, the G4 motifs in *FMR1* contribute to Fragile X syndrome [19]. Inherited disorders such as Werner and Bloom syndromes demonstrate compromised G4 resolution capabilities, highlighting their role in genomic stability [20]. Interestingly, the G4 elements discovered in *c-MYC* and *KRAS* genes regulate the biological function of these key oncogenes influencing cancer progression [21]. In particular, the telomeric G4 structures, which are crucial factors in cancer development, impact cellular senescence and immortalization [22]. These studies on G4s expand our understanding of their pathological significance and potential therapeutic applications. Notably, G4 structures have also been identified within viral genomes [23], such as SARS-CoV-2 [24,25], where their vital regulatory functions in viral processes and viral latency have been demonstrated [26]. These findings have paved new avenues for antiviral therapeutic strategies. Telomeres and the *C9orf72* gene are pivotal models for studying G4 structures due to their unique structural properties, biological significance, and direct implications in human diseases.

Moreover, extensive efforts have been made to target G4s as a therapeutic approach given their profound implication in diseases such as cancer and neurodegenerative diseases [27,28,29]. Early examples of G4-targeting ligands, such as quarfloxin and TMPyP4, demonstrated initial promise but faced significant limitations [21,30]. Recent developments have introduced more selective and viable G4 ligands, such as naphthalene diimides (NDIs), standing out as potent G4-binding ligands due to their ability to simultaneously target multiple G4s, exhibiting strong and selective anticancer activity [31]. Additionally, the tetra-substituted naphthalene diimide QN-302 is currently in clinical trials for pancreatic ductal adenocarcinoma (PDAC), highlighting its potential as a first-in-class G4-targeting therapeutic [32]. However, G4 conformations characterized by high precision are the key step of the therapeutic strategy. Therefore, the structure of G4s at high resolution is not only crucial for elucidating the pathogenic mechanisms of G4-related diseases, but also significant for the design of drugs targeting G4s in the treatment of such diseases. Specially, structural biology techniques, including high-resolution NMR spectroscopy and X-ray crystallography, combined with computational approaches have highlighted the structural diversity and dynamics of G4s, which has become crucial in developing novel therapeutic strategies for various G4-related pathologies.

Telomeric DNA sequences have a high propensity to form stable G4 structures under physiological conditions, and these G4 structures are well characterized, providing a robust model for studying G4 formation and stabilization [33]. Furthermore, the (GGGGCC)n repeat in *C9orf72* can form both unimolecular and multimeric G4s, which are highly stable and can drive the formation of biomolecular condensates [34,35,36]. The ability of these repeats to form G4s has been well documented, making them an excellent model for studying the structural and functional roles of G4s. In particular, small molecules that stabilize G4s in telomere and *C9orf72* have shown promise in inhibiting telomerase activity for cancer therapy and reducing the toxic effects of the repeat expansion in ALS/FTD models [27,28]. These studies demonstrate the potential of targeting G4s for developing drugs that can treat G4-related diseases. Therefore, G4s in telomere and *C9orf72* are particularly relevant models for studying G4s due to their unique structural properties, biological significance, and therapeutic potential. These models provide valuable insights into the role of G4 in both normal cellular functions and disease pathogenesis.

## 2. Human Telomeric G4 Structures

Telomeres, the repetitive DNA sequences located at the termini of eukaryotic chromosomes, are crucial to preserve chromosomal stability and genome integrity [37,38]. Extensive research has highlighted the significance of human telomeric DNA in cancer development and cellular aging mechanisms [39,40]. Telomeres in humans comprise tandem GGGTTA repeats spanning up to 10,000 base pairs, making them the richest sources of sequences that can form G4s [16]. In recent decades, the structural diversity and biological significance of human telomeric G4s have been extensively investigated. It is well known that the human telomeric d[GGGTTA]n sequence can fold into parallel, hybrid, and antiparallel structures including basket- and chair-type and (2 + 2) G4s [8,41,42,43,44,45,46,47,48,49]. We briefly summarized the reported telomeric G4 structures listed in Table 1. These diverse structural arrangements highlight the adaptability of telomeric sequences in response to different cellular environments and regulatory requirements.

**Figure 1:**
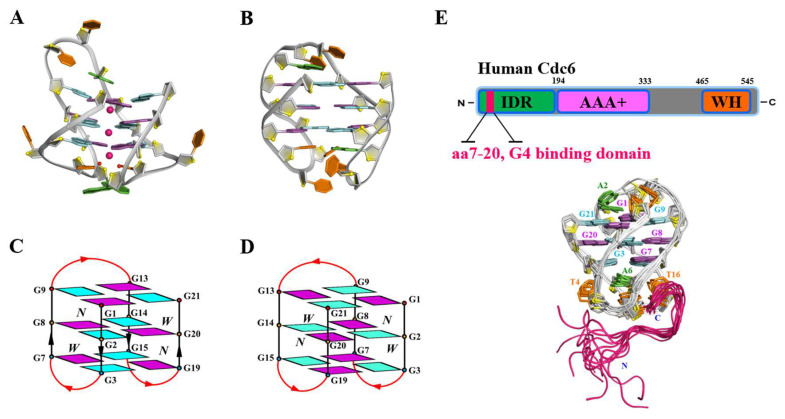
G4s formed by human telomeric DNA sequences and its complex with protein Cdc6. (**A**) The chair-type G4 structure formed by *htel21*_Br-8,20, d[GGGTTAG(^Br^G_8_)GTTAGGGTTAG(^Br^G_20_)G], in K^+^ solution (PDB: 6JKN). (**B**) The chair-type G4 structure formed by *htel21*T_18_, d[(GGGTTA)_2_GGGTTTGGG], in K^+^ solution (PDB: 5YEY). (**C**) The topology of *htel21*_Br-8,20. (**D**) The topology of *htel21*T_18_. (**E**) Schematic representation delineating the domain organization of human Cdc6 and the NMR structure of Cdc6 7–20 in complex with *htel21*T_18_.

Recently, we reported a novel chair-type G4 structure adopted by *htel21*_Br-8,20, d[GGGTTAG(^Br^G_8_)GTTAGGGTTAG(^Br^G_20_)G], two 8Br-dG substitutions at 8 and 20 positions of *htel21* (Figure 1A,C) [50]. This three-layer chair-type G4 features trinucleotide edgewise loops, with T5/T17 forming a water-mediated sandwich structure through K^+^ coordination and A6·A18 pairing, while a Hoogsteen A12·T10 pair caps the G-tetrad core, resulting in antiparallel strand arrangements. Interestingly, we also discovered that the same chair-type structure can be adopted by *htel21*T_18_, d[(GGGTTA)_2_GGGTTTGGG], a T substitution at A18 of *htel21* [51] (Figure 1B,D). This variant DNA adopts three-layer chair-type G4 featuring edgewise loops connected via reverse Watson–Crick A6·T18 pairing, with characteristic *syn*·*anti*·*syn*·*anti*glycosidic conformations in each G-terad. Strikingly, *htel21*_Br-8,20 and *htel21*T_18_, while sharing identical overall chair-type topology, exhibit reversed donor–acceptor directionalities in their individual G-tetrad layers (Figure 1C,D), demonstrating the subtle complexity of G4 structural arrangements.

Furthermore, we discovered that the human cell division cycle 6 (Cdc6) protein, a crucial DNA initiation factor, specifically recognizes the *htel21*T_18_ G4 structure [52]. The Cdc6 protein is composed of an N-terminal intrinsically disordered region (IDR), an ATPase-active AAA+ domain, and a C-terminal winged-helix domain (WHD). Through comprehensive biophysical analyses, we demonstrated that the N-terminal IDR (residues 7–20) of Cdc6 specifically binds to *htel21*T_18_. Our NMR structure of Cdc6 7–20 in complex with *htel21*T_18_ shows a hook-type conformation of the Cdc6 peptide, which is located at the bottom of *htel21*T_18_ (Figure 1E). The high-resolution NMR structure reveals the key residues including F14, P15, K16, R17, K18, L19, and S20, which are vital for Cdc6 in the recognition of G4. The biological significance of this interaction was confirmed through in vitro experiments demonstrating Cdc6 co-localization with G4 structures in cells [52]. These findings establish a previously uncharacterized mechanism by which Cdc6 recognizes specific G4 structures, potentially linking DNA replication regulation with G4-mediated genomic processes.

The study of telomeric G4 structures and their protein interactions have significant implications for understanding telomere biology and regulation. The discovery of the chair-type topology in telomeric G4 represents a significant advancement in studying G4 structural diversity. The high-resolution structure of the Cdc6-*htel21*T_18_ complex has revealed detailed molecular mechanisms of protein–G4 recognition, including specific base contacts and conformational adaptations, providing novel perspectives on the biological roles of telomeric G4 structures in DNA replication and cell cycle control. From a therapeutic perspective, these structural insights provide a foundation for designing small molecules or peptide-like compounds that could either stabilize specific G4s or modulate protein–G4 interactions, offering valuable insights for developing targeted drugs to treat diseases associated with telomeric G4s.

## 3. G4s Formed by *C9orf72* Hexanucleotide Repeats (G4C2)_n_

The abnormal expansion of a hexanucleotide, (GGGGCC)n (n from 30 up to thousands), in the first non-coding region of the *C9orf72* gene, is the predominant genetic factor in ALS and FTD [53]. These fatal neurodegenerative diseases, characterized by progressive neuronal dysfunction causing ultimate death, currently have no effective treatments available [54,55]. Accumulated molecular characterization indicates that *C9orf72* G4C2 repeat sequences, at both DNA and RNA levels, show remarkable structural polymorphism [56]. These sequences can adopt diverse secondary structures such as hairpin and G4 [57,58]. For example, the oligonucleotide d[(G4C2)_3_G4], serving as a minimal model for d(G4C2) repeats capable of unimolecular G4 formation, adopts two predominant G4 conformations alongside multiple minor species, all of which coexist in potassium solutions [59,60]. Most importantly, the G4 formation is notably associated with the toxicity of *C9orf72* G4C2 repeats in ALS/FTD, which is a gain-of-function mechanism through RNA foci formation sequestering RNA binding proteins (RBPs) [61]. Notably, RNA foci formed by *C9orf72* G4C2 repeats, characteristic pathological features of these disorders, are primarily composed of RNA G4s [17,62]. These structures appear to serve as nucleation sites for aberrant protein sequestration and aggregation, potentially initiating cascading cellular dysfunction [63]. Furthermore, *C9orf72* G4C2 repeat sequences form RNA condensates through multimolecular G4 structures. These RNA condensates play a critical role in disease pathology, potentially mediating neurotoxicity via liquid–liquid phase separation (LLPS) [36]. Moreover, *C9orf72* G4C2 creates an unstable folate-sensitive fragile site, FRA9A, in the genome. This fragile site is associated with abnormalities in DNA replication and repair, potentially leading to genomic instability and contributing to the pathogenesis of neurodegenerative diseases such as ALS/FTD [64].

**Figure 2:**
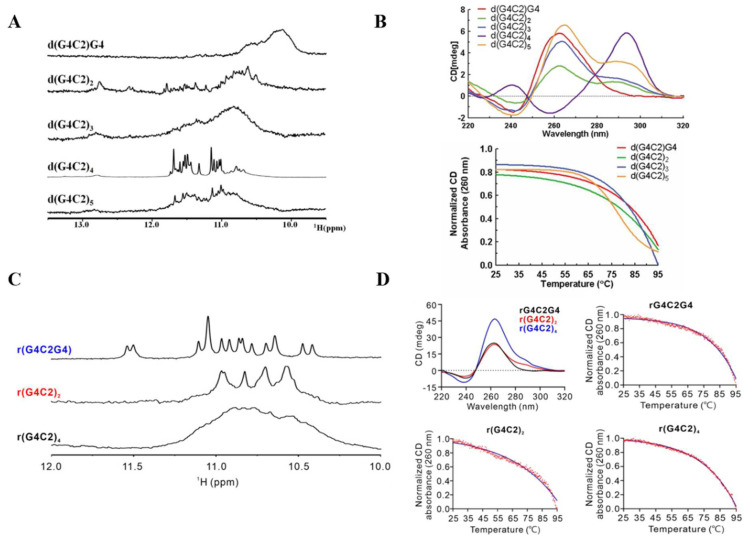
The characterization of different (G4C2)_n_ sequences in the *C9orf72* gene. (**A**) 1D ^1^H-NMR spectra of (G4C2)_n_ DNA sequences. (**B**) CD spectra of (G4C2)_n_ DNA. (**C**) 1D ^1^H-NMR spectra of (G4C2)_n_ RNA. (**D**) CD spectra of (G4C2)_n_ RNA.

Detailed structural analyses employing NMR and circular dichroism (CD) spectroscopy have revealed remarkable conformational diversity among *C9orf72* G4C2 sequences (Figure 2). *C9orf72* G4C2 DNAs demonstrate length-dependent topological preferences: d(G4C2)G4 adopts exclusively parallel conformations, while d(G4C2)_2_, d(G4C2)_3_, and d(G4C2)_5_ exhibit mixed topological arrangements [34] (Figure 2A,B). Notably, d(G4C2)_4_ uniquely assumes an antiparallel configuration, highlighting the sequence-specific nature of G4 folding patterns [34] (Figure 2A,B). In contrast to their DNA counterparts, *C9orf72* G4C2 RNAs adopt parallel G4s with different lengths of G4C2 repeats [56,57,65] (Figure 2C,D). This structural consistency in RNA G4s suggests distinct folding principles governing RNA versus DNA conformational preferences, potentially reflecting their divergent biological roles. The elucidation of high-resolution structure for these *C9orf72* G4C2 G4s is significant for multiple aspects of ALS/FTD pathobiology. These structural insights are crucial for understanding the molecular mechanisms underlying RNA-binding protein sequestration in ALS/FTD. Moreover, structural characterization provides essential foundations for de novo drug design, particularly enabling the development of structure-specific ligands or agents targeting distinct G4s.

**Figure 3:**
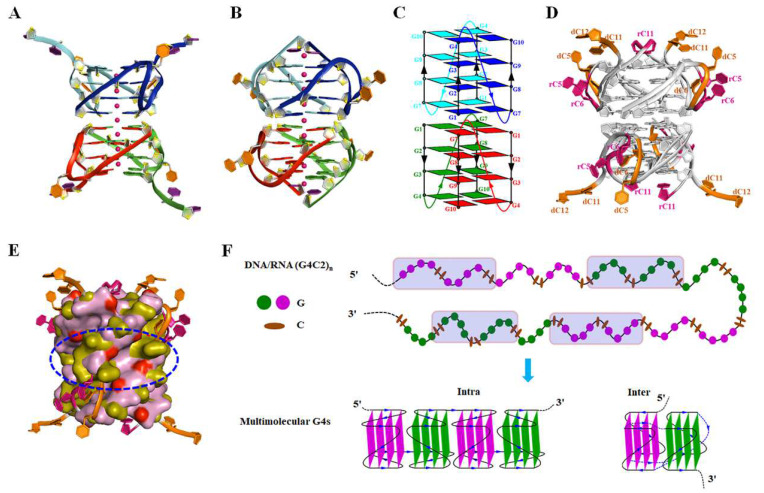
G4s formed by *C9orf72* HRE G4C2 DNA/RNA. Cartoon representation of tetrameric G4s formed by (**A**) d(G4C2)_2_ and (**B**) r(G4C2)_2_. (**C**) Topology of d(G4C2)_2_ and r(G4C2)_2_. Overlay of r(G4C2)_2_ (PDB:8X0S) and d(G4C2)_2_ (PDB:7ECH) G4 in (**D**) cartoon and (**E**) surface mode (the core of DNA in olive and RNA in pink), in which the 2′OH group of r(G4C2)_2_ is colored red and the CC bases are colored hot pink in RNA and orange in DNA, respectively. (**F**) Schematic diagram showing the formation of multimeric G4s by the DNA/RNA (G4C2)n, in which the sequential (G4C2)_4_ can form intramolecular G4s and the (G4C2)_2_ in rectangles colored pink can form intermolecular G4s.

In 2021, we reported the first crystal structure of d(G4C2)_2_ in K^+^ and Ba^2+^ solution, which adopts a parallel-stranded tetrameric eight-layer G4 composed of two dimeric G4 units (Figure 3A) [66]. The crystal structure of d(G4C2)_2_-Ba is obtained in the C2221 and F222 space groups, which adopt different arrangements of chains. The d(G4C2)_2_-Ba in the C2221 reveals that four chains assemble into two dimeric G4 units, which further stack in a 5′-to-5′ orientation to form a tetrameric G4 through π–π interactions. The d(G4C2)_2_-Ba in the F222 reveals asymmetric units consisting of three chains, which further organize into parallel-stranded tetrameric eight-layer G4s. Based on the structure of d(G4C2)_2_-Ba (F222), the d(G4C2)_2_-K structure in space group F222 was solved, which demonstrates isomorphous packing with similar structural features, differentiated primarily by their coordinating ions. In particular, our NMR studies demonstrated that the π–π stacking via 5′-to-5′ arrangement in crystal structure exists in solution [67]. Recently, we solved the first crystal structure of r(G4C2)_2_, which adopts a parallel eight-layer G4 (Figure 3B). The crystal structure indicated that two chains form parallel dimeric units that undergo 5′-to-5′ coaxial stacking with another symmetric dimeric unit to form an eight-layer parallel tetrameric G4 structure in K^+^ solution. This stacking is facilitated by π–π interactions (Figure 3B) [35].

Although the G4 structures of d(G4C2)_2_ and r(G4C2)_2_ adopt the same topology as depicted in Figure 3C, the G-core of these two G4s is nearly identical, with a root-mean-square deviation (RMSD) of ~2.42 Å. However, the C5 and C6 bases in these two G4s adopt different conformations and are located at distinct positions (Figure 3D). In the case of d(G4C2)_2_, the C6 base is situated within the medium groove of the G4 structure. Conversely, for r(G4C2)_2_, the C5 and C6 bases protrude outward, away from the G-core. Strikingly, the presence of the 2′OH groups and the 5′-to-5′ stacking mode in RNA leads to a shallower groove compared to DNA, which may represent unique and significant structural features for RBPs and ligands to recognize (Figure 3E).

Based on the G4 structures, we determined that both *C9orf72* DNA and RNA G4C2 sequences in vitro have the ability to form intramolecular four-layer parallel G4s [35,66]. These intramolecular structures sequentially stack together through 5′-to-5′ stacking, resulting in the formation of compact, higher-order G4 structures. Additionally, four neighboring G4C2 repeats along the same chain in different positions can associate to form intermolecular eight-layer parallel G4 structures. In these intermolecular structures, two four-layer parallel G4 units are connected by extended G4C2 repeats (Figure 3F).

Altogether, our structural findings are crucial for understanding the pathological mechanisms associated with the abnormal expansion of *C9orf72* DNA/RNA G4C2 repeats. Furthermore, the elucidated structures of *C9orf72* G4s demonstrate particular significance in multiple therapeutic aspects. First, they enable structure-based drug design approaches, facilitating the identification of small-molecule ligands with optimized binding properties for specific G4 structures. Second, these structures guide the development of therapeutic agents targeting distinct structures with specificity, potentially allowing targeted intervention at distinct stages of disease progression. Recent advances in structure-guided drug discovery have already yielded promising lead compounds demonstrating selective recognition of *C9orf72* G4 structures, which is significant for developing targeted therapies for ALS/FTD [27].

## 4. Conclusions and Future Perspectives

In recent years, studies on the biological role of G4s have validated the potential of designing specific ligands to target G4s in the treatment of disease. The structural diversity in telomeric G4s and *C9orf72* G4s particularly exemplifies the complexity and specificity requirements for targeted therapeutic development. The principal challenge in G4-targeted therapeutics is the selective recognition of specific structural motifs. This requirement of specificity highlights the importance of high-resolution structural characterization for each identified G4. Detailed structural information enables rational approaches to design novel ligands with optimized binding properties and reduced off-target effects. The expanding recognition of G4 involvement in human pathologies, from neurodegenerative disorders to cancer, positions structural studies at the forefront of therapeutic innovation. Future investigations focusing on G4 structures and interaction with cellular factors promise to advance our understanding of disease mechanisms and therapeutic strategies. This evolving field presents exciting opportunities for structure-guided drug discovery and the development of novel therapeutic modalities targeting specific G4 structures in various diseases.

## Figures and Tables

**Table 1:** Telomeric G4 structures.

PDB ID	Sequence	Structure Type	Solution Conditions	Method	Structure
143D	AGGG(TTAGGG)_3_	antiparallel basket-type	Na^+^	NMR	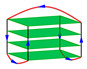
1KF1	d[(AGGGTT)_3_AGGG]	parallel	K^+^	XRD	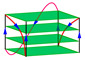
1K8P	d[^Br^UAGGG^Br^UTAGGGT]	parallel	K^+^/Na^+^	XRD	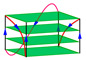
2GKU	d[TTGGG(TTAGGG)_3_A]	hybrid	K^+^	NMR	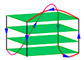
2HY9	d[AAAGGG(TTAGGG)_3_AA]	hybrid-1	K^+^	NMR	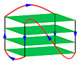
2JPZ	d[(TTAGGG)_4_TT]	hybrid-2	K^+^	NMR	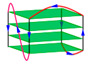
2JSM	d[TAGGG(TTAGGG)_3_]	hybrid-1	K^+^	NMR	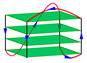
2JSL	d[TAGGG(TTAGGG)_3_TT]	hybrid-2	K^+^	NMR	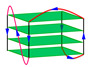
2JSK	d[TAGGGTTAGGGTTAG(^Br^G)GTTAGGG]	hybrid-1	K^+^	NMR	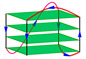
2JSQ	d[TAGGGTTAGGGTTA(^Br^G)GGTTAGGGTT]	hybrid-2	K^+^	NMR	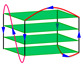
2KF8	d[(GGGTTA)_3_GGGT]	basket-type	K^+^	NMR	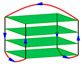
2KF7	d[GGGTTA(^Br^G)GGTTAGGGTTAGGGT]	basket-type	K^+^	NMR	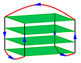
2KKA	d[(AGGGTT)_2_AIGGTTAGGGT]	basket-type	K^+^	NMR	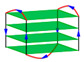
5YEY	d[(GGGTTA)_2_GGGTTTGGG]	chair-type	K^+^	NMR	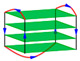
6JKN	d[GGGTTAG(^Br^G)GTTAGGGTTAG(^Br^G)G]	chair-type	K^+^	XRD	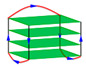

NMR: Nuclear magnetic resonance, XRD: X-ray diffraction.
